# Delayed primary closure in open abdomen with stoma using dynamic closure system

**DOI:** 10.1186/s40064-015-1316-9

**Published:** 2015-09-17

**Authors:** Juan Manuel Suarez-Grau, Juan Francisco Guadalajara Jurado, Julio Gómez Menchero, Juan Antonio Bellido Luque

**Affiliations:** General Hospital of Riotinto, Minas de Riotinto, Huelva, Spain

**Keywords:** Open abdomen, Surgery, ABRA, VAC, Abdominal wall, Closure, Compartimental syndrome

## Abstract

**Background:**

The situation of abdominal sepsis secondary to colonic perforation sometimes forces treat the patient with multiple interventions in the open abdomen (OA) context. Correct management of OA is important to restore the patient’s clinical situation and to avoid further complications of the abdominal wall. Delayed primary closure of the abdomen using a dynamic and progressive traction is a relatively new technique for treating the OA.

**Case presentation:**

We report the case of a 50 year old woman with history of malnutrition and chronic obstructive pulmonary disease, affects for an OA after several surgical interventions. Two previous interventions (right colectomy, ileostomy and laparotomy with Bogotá bag) for disseminated peritonitis and abdominal compartment syndrome were performed. Six days after the Bogota bag the of the dynamic closure system ABRA^®^ system was placed to delayed primary closure of the abdomen with excellent result results of the contingency of the abdominal wall.

**Discussion:**

The most common technique in the current management of OA is the placement of vacuum-assisted closure or the use of a mesh. These systems generally require several operations to restore the integrity of the abdominal wall. However, the dynamic closure of the abdominal wall makes it possible to restore it into the same process.

**Conclusions:**

ABRA system allows delayed primary closure of the abdominal wall in an OA by sepsis secondary to colonic perforation. The stoma was not a problem with this technique. The final closure of the abdomen was at 16 days after the ABRA placement. The abdominal wall has not alterations in the follow up after 3 years.

## Background

The management of open abdomen (OA) should be life-saving in abdominal Compartimental syndrome, trauma, severe secondary peritonitis, postoperative abdominal wound dehiscence. Various temporary abdominal closure (TAC) techniques have been described in treatment of the open abdomen. Vacuum-assisted closure (VAC) abdominal dressing is the most common therapy in the Intensive Care Unit (ICU) for these patients, but this technique requires the corrections of posterior problems for the reconstruction of the integrity of the abdominal wall and the skin.

After temporary abdominal closure, the abdominal fascia must be closed primarily. The first goal is delayed primary fascial closure; however, other surgeons use mesh and/or granulation tissue with split-thickness skin grafting to close the abdominal wound.

The Abdominal Re-approximation Anchor system (ABRA^®^, Canica, Almonte, Ontario, Canada) is a novel technique based on dynamic elastic closure. It was designed specifically for the delayed closure of the OA. In the case report, we describe the use of this abdominal re-approximation technique in an OA patient by Compartimental syndrome due to peritonitis after two colonic interventions.

## Case report

We present a the case of a 50 year old woman (history of malnutrition, chronic obstructive pulmonary disease) accepted in the Digestive and Surgery Division due to an intestinal obstruction of 3 days. The patient underwent emergency surgery for intestinal obstruction, with removal of impacted bezoar in the ileocecal valve. After 48 h, the patient started in sepsis by intestinal suture dehiscence. Right hemicolectomy was performed urgently with cleaning of the peritoneal cavity. After 72 h of operation, in the ICU, the patient situation turn to worse by a suture dehiscence of the anastomosis, requiring a new emergency surgery. Due to septic state by a colonic dehiscence and disseminated peritonitis, and the inability to perform anastomosis, colectomy and terminal ileostomy. A Bogotá bag laparostomy was precised due to the abdominal Compartimental syndrome (intra-abdominal pressure index of 25, intraabdominal pressure measurements was done intravesically).

**Fig. 1:**
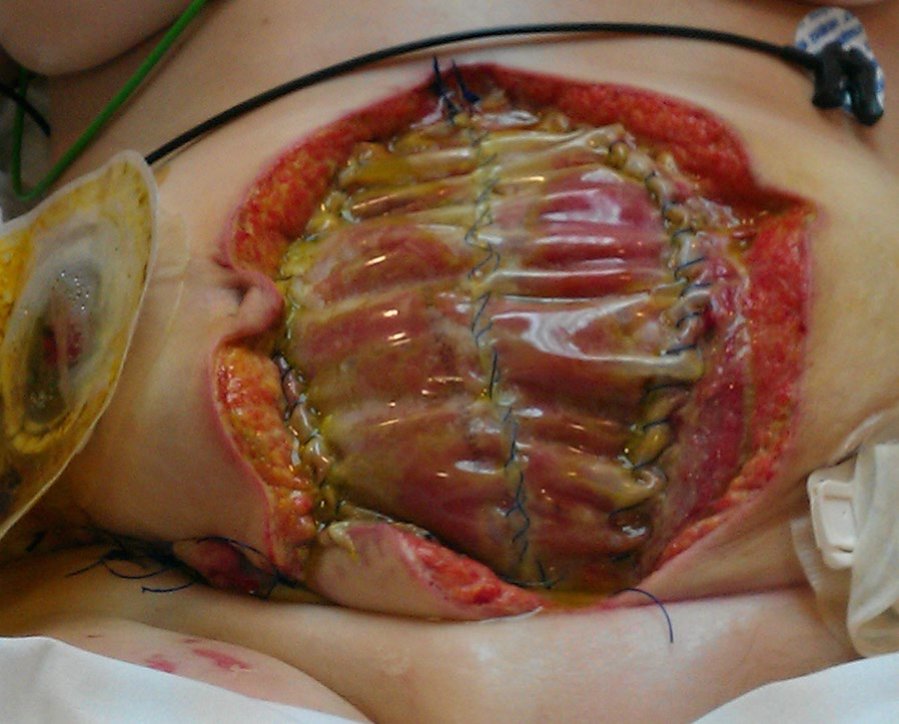
Bogotá bag

**Fig. 2:**
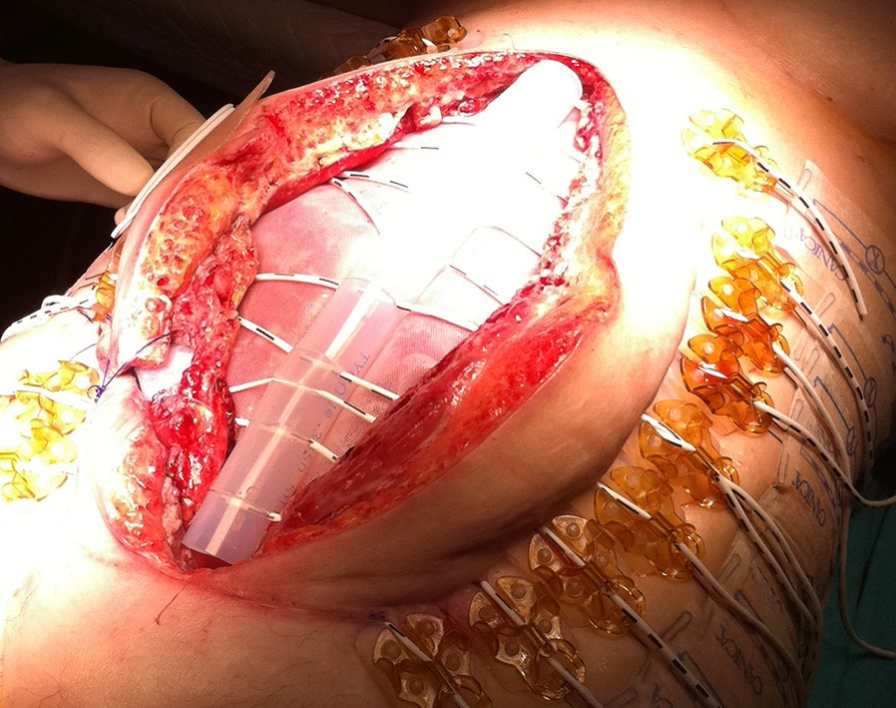
Dynamic closure system (ABRA^®^)

**Fig. 3:**
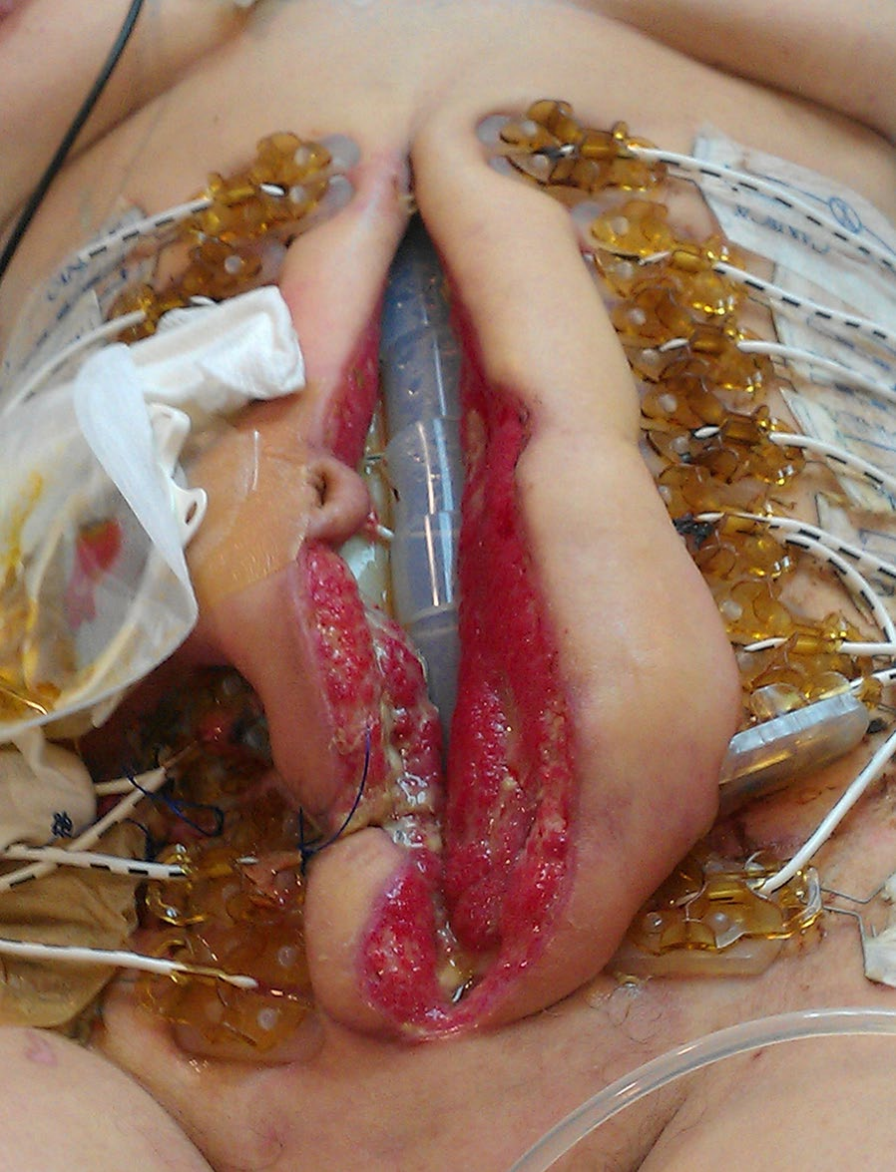
Total approximation of the borders of the fascia and skin before primary closure

In the ICU the APACHE II score was in the first week 15 (first week range 8–17) and the Mannheim peritonitis index was 34 (first week range 28–36). OA scoring by Bjórcket al was 2B (Fig. 1). After stabilization in the ICU the Bogotá bag was removed 6 days later, and we placed dynamic closure system (ABRA^®^) (Fig. 2). IAP measurements were performed in the patient during ABRA treatment in the ICU, but no pathologic values were recorded. The measurement of the abdominal wound was: 15 cm wide and 27 cm length. The ileostomy in the lower right quadrant functioned normally. The fascia was approximated one cm per day. Sixteen days later we proceeded to the primary closure of abdomen (Fig. 3).

**Fig. 4:**
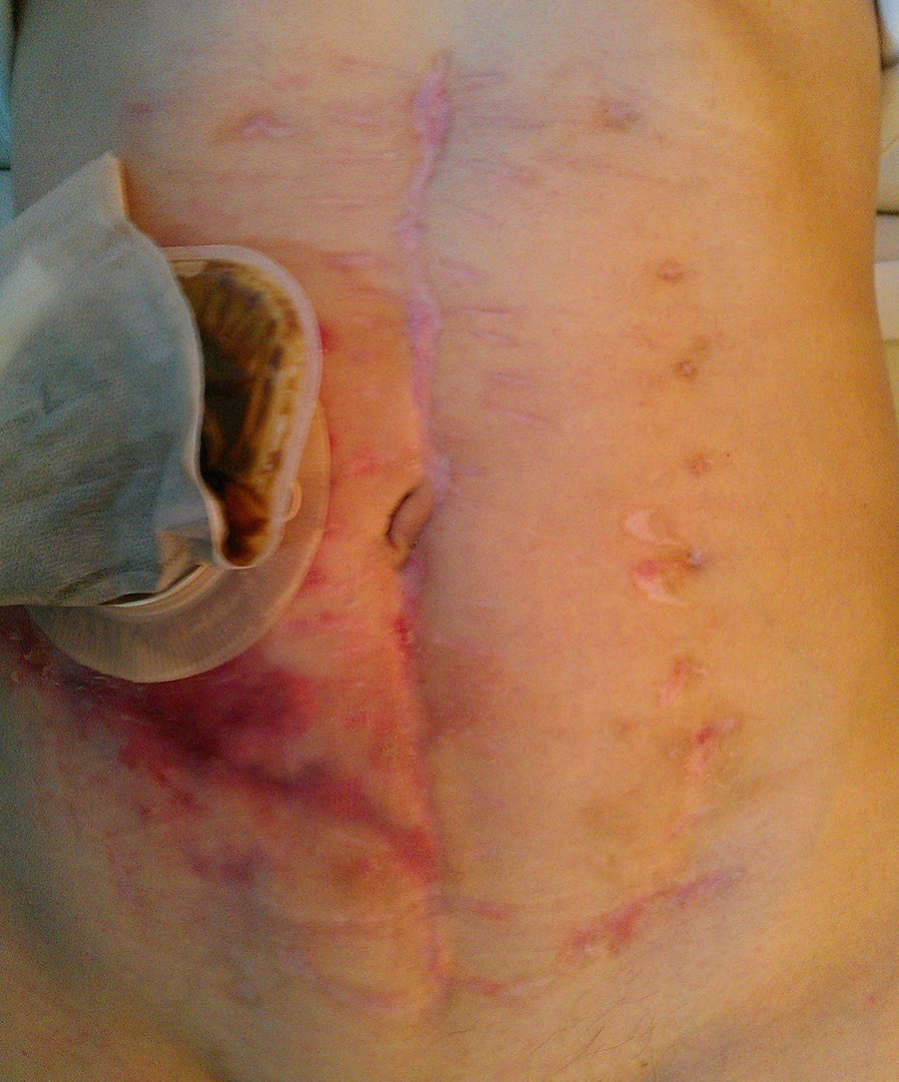
Final appearance of the abdomen

The patient was discharged from the ICU after 2 days since the primary closure. Finally the patient was discharged a week from the Hospital. There was no dehiscence of skin and wound had healed properly in the follow up after 3 years (Fig. 4).

## Discussion

Patients with an OA are critically ill and have a high risk of developing major complications: multiple organ dysfunction syndrome, enterocutaneous fistula, intraabdominal abscess, and abdominal wall hernia (around 25–35 %) (D’Hondt et al. 2011; Brandl et al. 2014; Verdam et al. 2011). We have several techniques for the TAC: Negative Pressure Wound Therapy (NPWT) VAC, Vacuum pack, Zipper, Artificial burr, Mesh/sheet, Silo, Dynamic retentions sutures, etc (Suliburk et al. 2003; Tremblay et al. 2001; Wittmann 2000; Cuesta et al. 1991).

The TAC using VAC is the most used technique in OA. The availability and preference for these techniques seems to have evolved during the past 30 years. At present, vacuum based techniques seem to be popular because 85 % of the studies published since 1998 describe a vacuum technique (D’Hondt et al. 2011; Suliburk et al. 2003). The high closure rate and the low complications rate have allowed this technique the most accomplished. These methods allow decompression of the abdomen, sequential lavage, and debridement, and they do not damage the midline fascia. VAC therapy has the additional advantage of evacuating the inflammatory exudate (Cuesta et al. 1991; Boele van Hensbroek 2009; Howdieshell et al. 2014; Smith et al. 1992).

Despite this advantages of the technique, many OA patients often develop large and debilitating hernias of the abdominal wall that require complex repair surgery at a later stage (around 35 % of patients) (Brandl et al. 2014). Other disadvantages of the NPWT are the need to carry the portable pump ant the cost of the devices; these systems are more expensive than others TAC (D’Hondt et al. 2011; Smith et al. 1992; Salman et al. 2014).

Generally the closure time of the wound take several weeks, with a high hospital stay rates (Olona et al. 2015).

These are the main reasons for the use in selected cases of the ABBRA system: the reduction of the hospital stay (Olona et al. 2015), the cost of the system, and the prevention of the ventral hernia after OA infected (Verdam et al. 2011; van Hensbroek 2009; Salman et al. 2014). In addition the traction provided by the ABRA system is dynamic, can continuously be adjusted, and permits both expansion and retraction without damaging the fascia (Table 1).

**Table 1:** Summary of pros and cons of the ABRA system in open abdomen

ABRA system delayed closure system	
Pros	Cons
ABRA abdominal wall closure can restore lost abdominal domain and achieve complete repair of the musculofascial support of the abdominal wall, achieving primary closure. The sutures can be tightened at sequential dressings, preventing fascial retraction	ABRA abdominal wall closure requires two to three interventions in operation room under general anesthesia
No skin grafts required when using ABRA	ABRA system need a normal skin of 5–6 cm around the abdominal wound (in order to place the elastomers)
Reduction in the numbers of days to any closure	The possibility to need to be used in conjunction with another dressing system
The changes of VAC system and time in nurse and surgery cares are more expensive than ABRA system	There is no active removal of the fluids. So if the peritonitis is still active a dressing system with 24–48 h re-interventions could be the best option

We consider this system a useful tool in the treatment of septic patients with an open abdomen.

## Conclusions

Abdominal closure was performed without complications of surgical wound and no evidence of incisional hernias 3 years after the intervention. Dynamic closure systems allow progressive delayed primary closure of the secondary open abdominal sepsis abdominal wall of colonic origin, and the presence of stoma is not a contraindication for it use.
